# The functional role of locus coeruleus microglia in the female stress response

**DOI:** 10.1038/s41380-025-02971-9

**Published:** 2025-04-05

**Authors:** Cora E. Smiley, Brittany S. Pate, Samantha J. Bouknight, Evelynn N. Harrington, Aaron M. Jasnow, Susan K. Wood

**Affiliations:** 1 Department of Pharmacology, Physiology, and Neuroscience; University of South Carolina School of Medicine, Columbia, SC 29209 US; 2 WJB Dorn Veterans Administration Medical Center, Columbia, SC 29209 US; 3 University of South Carolina, Department of Exercise Science, Columbia, SC 29209 US; 4 USC Institute for Cardiovascular Disease Research, Columbia, SC 29209 US

**Keywords:** Neuroscience, Biological techniques

## Abstract

Neuropsychiatric disorders that result from stress exposure are highly associated with central inflammation. Our previous work established that females selectively exhibit heightened proinflammatory cytokine production within the noradrenergic locus coeruleus (LC) along with a hypervigilant behavioral phenotype in response to witnessing social stress. Notably, ablation of microglia using pharmacological techniques prevents this behavioral response. These studies were designed to further investigate the impact of stress-induced neuroimmune signaling on the long-term behavioral and neuronal consequences of social stress exposure in females using chemogenetics. We first characterized the use of an AAV-CD68-G_i_-DREADD virus targeted to microglia within the LC and confirmed viral transduction, selectivity, and efficacy. Clozapine-n-oxide (CNO) was used for the suppression of microglial reactivity during acute and chronic exposure to vicarious/witness social defeat in female rats. Chemogenetic-mediated inhibition of microglial reactivity during stress blunted the neuroimmune response to stress and prevented both acute and long-term hypervigilant behavioral responses. Further, a history of microglial suppression during stress prevented the heightened LC activity typically observed in response to stress cues. These studies are among the first to use a chemogenetic approach to inhibit central microglia in vivo and establish LC microglia as a key driver of the behavioral and neuronal responses to social stress in females.

## Introduction

Exposure to social stressors is a risk factor for many neuropsychiatric disorders including post-traumatic stress disorder (PTSD) [1, 2]. While up to 90% of the general population reports experiencing a traumatic event that is often psychosocial in nature [3], around 15% of these people will go on to develop conditions such as PTSD [4]. Interestingly, while men report greater levels of overall trauma exposure, women develop PTSD at twice the rate of males [5]. Further, there are different symptom patterns between the sexes with the hyperarousal and hypervigilance aspects of PTSD symptomology [6] observed to a higher degree in females [7, 8]. These symptoms are marked by overall higher levels of arousal and contextual and environmental cues that are reminiscent of the initial traumatic event are capable of inducing a hypervigilant state [9]. Early emergence of hyperarousal and hypervigilance are also associated with less symptom improvement over time and worse treatment outcomes when compared to traumatized individuals that did not exhibit this symptom profile [10, 11]. Thus, there may be inherent neurobiological factors that promote hypervigilance and are responsible for the 2-3x greater risk of developing stress-related disorders in females [8].

One brain region highly involved in stress responses and hypervigilance is the noradrenergic locus coeruleus (LC) [8, 12–15]. This hindbrain region includes tonically active norepinephrine (NE) producing cells that can phasically respond to stimuli, resulting in escalated NE release to downstream effector regions [14, 16]. Exposure to stress increases phasic activity within this region, and LC hyperactivity is evident in patients with PTSD [17] to directly promote hypervigilance [13, 18]. Therefore, further research into the underlying mechanisms responsible for stress-induced increases in LC-NE activity is essential for understanding the role of this system in the hypervigilance-related behavioral dysfunction observed following traumatic stress exposure. Importantly, the LC has been shown to be highly regulated by neuroimmune signaling, with proinflammatory cytokines selectively administered within this region leading to increased neuronal firing [19, 20] which may contribute to increases in LC activity observed in response to stress. In accordance with this hypothesis, PTSD, among other stress-induced neuropsychiatric disorders, is associated with increases in inflammatory factors in the brain and periphery [21–23]. Further, neuroimmune signaling is associated with altered behavioral responding following stress in rodent models [24, 25] that mimics the psychiatric dysfunction found in clinical populations [26], especially in females [27, 28]. In fact, females display exaggerated responses to immune stimulation, with lipopolysaccharide (LPS) injections evoking greater inflammatory, noradrenergic, and cortisol responses in women which were associated with worsening mood symptoms that were not observed in men [26, 29]. Overall, these data support the hypothesis that the predisposition to stress-related neuropsychiatric disorders in females may be due to an underlying sensitivity to the neuroimmune impact of stress.

Despite accumulating evidence that suggests a role for neuroimmune signaling in heightened stress responding in females, studies examining a specific role for microglia are sparse, largely due to a lack of effective tools in the field. Our previous studies using clodronate, a compound designed to pharmacologically ablate microglial cells, support the hypothesis that microglia within the LC play a major role in the female response to social stress [25]. Therefore, by utilizing chemogenetics to transiently suppress microglial reactivity during social stress exposure, these studies were designed to further explore the role of stress-evoked neuroimmune signaling in the acute and long-term behavioral consequences associated with hypervigilance in females. Further, the impact of prior intra-LC microglial inhibition on neuronal activity was assessed in response to stress cues to determine how microglia shape future stress context-dependent neuronal activation. DREADD viruses designed to chemogenetically inhibit microglia have been previously characterized for use in the spinal cord [30, 31] and, through G-protein coupled receptors (GPCRs), can prevent microglial reactivity and subsequent release of proinflammatory cytokines [32–35]. Initial reports using these viral constructs conducted in vitro experiments in which microglial cells were cultured and transfected with either hM3D_q_ or hM3D_i_ viral constructs before treatment with LPS and CNO. These studies determined that G_i_-mediated suppression of LPS-induced microglial reactivity using CNO reduced the RNA levels of eight major inflammatory signaling molecules, including the proinflammatory cytokine IL-1β, while these factors were increased when G_q_ receptors were stimulated [30]. These effects were replicated in vivo using rat models with DREADD administration in the spinal cord [30, 31]. Thus, the present experiments used these chemogenetic techniques in female rats to modulate microglial reactivity during acute and repeated psychosocial stress to assess the short- and long-term behavioral impact of stress-induced neuroimmune signaling in the LC.

## Materials and methods

### Animals

Female (~200 g, 9 weeks, witnesses/controls) and male Sprague-Dawley rats (~250 g, intruders) were obtained from Charles River (Durham, NC) while male Long-Evans retired breeders (600–800 g, residents) were obtained from Envigo (Dublin, VA). All rats were housed individually in standard polycarbonate cages. Female rats that underwent stress and all males were housed in a separate but adjacent room from control females. Rats were maintained on a 12-hr light/dark cycle (on at 0700), received *ad libitum* access to food and water, and were housed in cages containing Teklad sani-chip bedding (Envigo). Studies were approved by the University of South Carolina’s Institutional Animal Care and Use Committee and maintained adherence to the National Research Council’s Guide for the Care and Use of Laboratory Animals.

### Study design

**Fig. 1:**
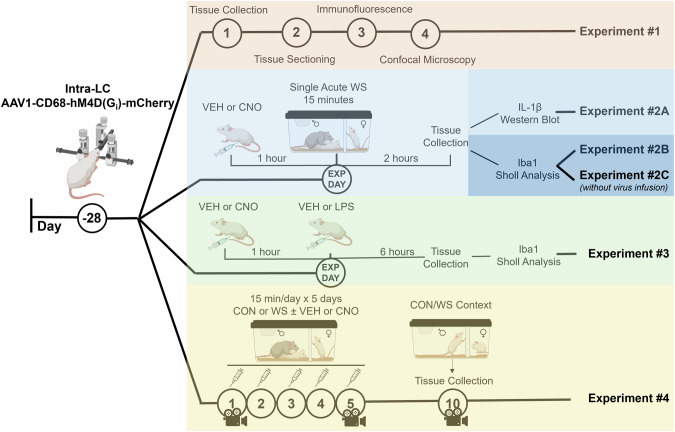
Experimental Design. All studies incorporating the DREADDs virus started with stereotaxic surgery to inject 500 nL of the AAV1-CD68-hM4D(Gi)-mCherry into the locus coeruleus (LC) guided by electrophysiological recordings for precise LC targeting, followed by a four-week recovery to allow for viral expression prior to study initiation. One separate subset of rats (experiment 2C) remained surgically naïve with no viral injection to test the non-specific effects of clozapine n-oxide (CNO). *Experiment #1*: brain tissue was sectioned to collect the LC and stained for Iba1 (microglia), GFAP (astrocytes), and NeuN (neurons) to determine viral selectivity to microglia. *Experiment #2*: rats were injected with either vehicle or CNO one hour prior to a single acute exposure to witness stress (WS) and tissue was collected two hours later for either western blot (*2A*) or microglial morphology (*2B*) analysis. A subset of rats underwent the same experimental protocol as Experiment 2 but received CNO administration in the absence of viral administration in the LC (*2C*). *Experiment #3*: vehicle or CNO was administered one hour prior to a peripheral injection of lipopolysaccharide (LPS) and brain tissue was collected six hours later for microglial morphology analysis. *Experiment #4*: rats were exposed to repeated WS with vehicle or CNO injections occurring one hour prior, followed 5 days later by an acute exposure to the WS cues and context in the absence of VEH/CNO administration, after which brain tissue was collected for immunohistochemical analysis.

There were four main experiments completed to characterize the use of chemogenetic techniques for the site-specific manipulation of microglial activity in the rat hindbrain and to determine the role of LC microglia in the behavioral and neuronal responses to social stressors in females (Fig. 1). All rats were randomly assigned to treatment groups and power analyses were completed based on variability from previous experiments to establish the group sizes included across the following four main studies. Experiment #1 (*n* = 8): determine the cell type selectivity of viral transduction into microglia, astrocytes, or neurons when pAAV1 CD68-hM4D(G_i_)-mCherry virus was infused into the LC. Experiment #2A (*n* = 22 total rats; *n* = 7-8/group, CON+VEH vs. WS+VEH vs. WS+CNO): assess how DREADD activation affects microglial-mediated cytokine production in response to stress. Experiment #2B (36 total rats; *n* = 6/group for behavior, 4/group for microglial stereology): determine the impact of chemogenetic suppression of microglial reactivity on stress-induced behavioral responding and LC microglial morphology. Experiment #2C (*n* = 28, 7/group for behavior, 4/group for microglial analysis): establish behavioral responses to acute stress and microglial ramification characteristics in the absence of viral infusion and in the presence of CNO treatment to determine any off-target drug effects. Experiment #3 (*n* = 16 total rats; *n* = 4/group, VEH/LPS+VEH/CNO): evaluate the effects of CNO-mediated microglial silencing in response to LPS on microglial morphology in the LC. Experiment #4 (34 total rats; *n* = 8-9/group): determine the effects of acute and repeated vicarious/witness defeat stress ± DREADD-mediated suppression of microglial responding on behavioral and neuronal responses to stress cues.

### Surgical procedures and viral infusion

All experiments, excluding Experiment #2C, commenced with stereotaxic surgery to infuse a pAAV1 CD68-hM4D(G_i_)-mCherry virus (Addgene viral prep #75033-AAV1 from Bryan Roth, Addgene, Watertown, MA) into the LC for selective targeting of microglial cells (as established in [30, 31, 36]). All rats were injected with the active virus and systemic vehicle injections were used in parallel with CNO administration to control for viral activation.

Rats were anesthetized using isoflurane (5% for induction, 2-3% maintenance) and electrophysiological recordings were used for precise identification of the LC. The nose was angled down 10° and, starting at A/P: -4.0; M/L: ± 1.2, D/V: -4.5 (mm from bregma and the surface of the dura), a tungsten coated electrode was lowered through the tissue while an oscilloscope relayed neuronal firing patterns. Once the LC was located, these coordinates were used to lower a microsyringe bilaterally (Hamilton, Franklin, MA) to inject 500 nL of the virus (100 nL/minute + 5 min pressurization). Postoperative care consisted of saline (10 mL/kg, s.c.), flunazine (0.25 mg/kg, s.c.), and supplemental nutrition (Bacon Softies, Bio-Serv) as needed. Rats were allowed 28 days prior to study initiation to allow for viral expression during which they received i.p. saline injections 3x/week for injection habituation. Viral localization to the LC was confirmed with microscopic visualization of 30-micron tissue slices and injections sites for each treatment group are displayed in the supplement (Figure S1). Although electrophysiological confirmation of localization during surgery greatly reduces the number of off-target injections, one animal was discovered to have unilateral viral misplacement and was eliminated from analysis.

### Drug information

Rats were administered water soluble clozapine-n-oxide (CNO) (1 mg/kg, HelloBio, Princeton, MA) or vehicle (0.9% saline) intraperitoneally (i.p.) one hour before the start of stress/control either once (acute stress) or for five days in a row (repeated stress). Lipopolysaccharide was diluted in saline to 3 µg/µL and was administered at a dose of 30 µg/kg one hour following the CNO pretreatment in Experiment #3.

## Behavioral measurements

### Witness stress or control handling

Female rats were exposed to witness stress paradigms (WS) as previously described [24, 25, 37, 38]. Briefly, a female was placed behind a clear perforated Plexiglas partition in the cage of a male Long-Evans retired breeder (resident) to allow for the transfer of visual, olfactory, and auditory cues of the social defeat with the male intruder without physical access (described in detail in [24]). Female rats were either exposed to a single 15-minute acute stressor (Experiment #2) or this paradigm was completed once/day for a total of five days (Experiment #4). Witnesses were paired with the same male intruder rat for daily WS, but the resident was novel each day to maintain attack behaviors. Control rats (CON) were briefly handled and returned to their home cage where behaviors were recorded. Previous experiments from our lab established that females exposed to the resident’s cage behind the partition with the intruder on the opposite side [24] or in absence of the intruder [37], do not exhibit behavior that differs from rats handled and returned to their home cage. Sessions were video recorded on the first and fifth days of exposure to allow for retrospective blinded manual scoring of stress-evoked behaviors. Behaviors including burying, freezing, and rearing were quantified according to our previous publications [24, 25, 37].

### Control or witness stress context exposure (CON/WS CXT)

WS CXT consisted of placing the female rat behind the partition in the residents’ cage with their paired intruder opposite but in the absence of the resident to expose the females to the sensory cues and environment in which they originally experienced social stress. Previous studies from our lab have determined that a history of stress exposure leads to increased burying in the WS CXT and this paradigm serves as a method of translationally measuring hypervigilant behaviors [37]. Behaviors were scored identically to those assessed during WS and were compared to CON rats that were handled and recorded in their home cages.

### Euthanasia and tissue collection

All euthanasia procedures followed the guidelines set forth by the American Veterinary Medical Association (AVMA). Following deep anesthetization with isoflurane, confirmed by a lack of reflexive responding to a toe pinch, rats were either transcardially perfused with 0.1 M phosphate buffered saline (PBS) followed by 4% paraformaldehyde (4% PFA prepared in 0.1 M PBS) and rapidly decapitated for tissue collection. Perfused brains were extracted, post-fixed in 4% PFA for at least 2 h, and saturated with 20% sucrose + 1% azide upon which they were flash frozen using ice cold isopentane and stored at -80 °C until further analysis. Non-perfused rats were euthanized by rapid decapitation following isoflurane anesthetization, and fresh tissue was collected and flash frozen prior to dissection.

## Brain analyses

### Immunohistochemistry

Perfused brains were sliced at 30 microns on a cryostat and LC slices were collected, ensuring that the same anatomical level was selected between animals. Tissue slices were blocked for 30 min in 0.9% hydrogen peroxide + 4% Triton-X 100 (TX) in 0.1 M PBS and incubated in the following 1° antibodies overnight at room temperature: anti-Iba1 1:1000 (rabbit, FujiFilm Wako Chemicals #019-19741), anti-cfos 1:500 (rabbit, Millipore #ABE-457), and anti-TH 1:2500 (mouse, Millipore #MAB-5280). The following day, tissue slices were placed in a 2-hour incubation in secondary antibody that corresponds with the species of the primary: biotinylated anti-rabbit 1:200 (Vector Labs #BA-1000) or biotinylated anti-mouse 1:1000 (abcam #ab208001). Tissue was incubated in avidin biotin complex (Vectastain Elite ABC Kit, Vector Laboratories) for 1 h and stained in diaminobenzidine (DAB) or metal enhanced DAB (Vector Laboratories) for double labeling experiments. The DAB development times were optimized for each antibody (1 min and 30 s for Iba1, 2 min for cfos, and 15 s for TH). Tissue was mounted on slides (SuperFrost), dehydrated, and coverslipped with Permount (Fisher Scientific). Slides were imaged using a Microbrightfield microscope and traced by Neurolucida software (MBF Biosciences) for quantification of microglial morphological characteristics or a Nikon E600 microscope and counted for cfos/TH+ cells in Image J (NIH) by an experimenter blinded to the treatment conditions.

### Immunofluorescence (IF)

Brain slices were blocked for 30-minutes in a buffer containing 2% NDS, 50 mM glycine, 0.05% Tween-20, 0.1% TX, and 0.01% bovine serum albumin (BSA) in 0.1 M PBS [39]. Sections were then placed into primary antibody overnight at 4 °C for either anti-Iba1 1:1000 (rabbit, FujiFilm Wako Chemicals #019-19741), anti-NeuN 1:500 (mouse, Millipore #MAB-377), or anti-GFAP 1:500 (rabbit, Cell Signaling #12389T) primary antibody. Subsequently, tissue was incubated in the secondary antibody (anti-rabbit Alexa-Fluor 488, 1:500, abcam #150073 or anti-mouse Alexa-Fluor 488, 1:500, abcam #ab1501113) before mounting onto slides (SuperFrost) and coverslipped with Prolong Diamond Media (Thermo Fisher). Slides were imaged on a confocal microscope (Leica Stellaris).

### Western blot analysis

LC tissue punches were collected for Western Blot analysis of IL-1β protein concentration. Flash frozen brains were sliced on a cryostat (Leica) starting at the brain stem, until reaching the most posterior point of the LC, at which time a tissue biopsy punch (1 mm in diameter, 1 mm depth) was used to selectively dissect out the LC. Post punch slices were collected and stained with Neutral Red to confirm that all LC cells were removed (Supplemental Figure S2). Tissue was homogenized according to the methods presented in previous publications [25, 37]. Supernatant was collected and assessed for protein concentration using a Pierce Bicinchoninic Acid (BCA) assay (Thermo Scientific, Rockford, IL) per the manufacturers protocol. Samples containing 20 µg of protein were aliquoted for Western Blotting following previously published protocols [37]. Western Blots were probed for the antibodies IL-1β (1:200, goat, R & D Systems #AF-501-NA) and the housekeeping protein GapDH (1:1000, mouse, Santa Cruz #sc-365062) overnight followed by secondary antibodies (IR-Dye anti-rabbit 800 nm (LI-Cor #926–32213) and IR-Dye anti-mouse 680 nm (LI-Cor #962–68072) diluted 1:20,000) for 1 h. Membranes were imaged on a LI-Cor Odyssey scanner (LI-Cor Biotechnology, Lincoln, NE), and protein expression was quantified using LI-Cor Image Studios Software with normalization to GapDH. Full Western Blot images are provided within the supplement (Figure S3).

### Analysis of microglial ramification characteristics

Microglial morphology was assessed using Neurolucida Explorer software to trace individual microglia cell bodies and their projections. LC tissue sections labeled with Iba1 and stained with DAB were imaged at 100x magnification on a Microbrightfield microscope equipped with Neurolucida software (MBF Biosciences) for cellular tracing and reconstruction. Four rats from each treatment group were randomly chosen for morphological analysis with four LC tissue sections assessed per animal and five cells traced per section. For each rat, the data for each tissue section was averaged to allow for an *n* = 20 cells/animal and *n* = 4 animals per group. Microglial reconstructions from Neurolucida were imported into Neurolucida Explorer software for automated quantification of key morphological features including the cell body area, the number of projection endpoints, the longest projection, the total length of all projections, and the Sholl curve which was established based on the number of projections intersecting increasing diameters from the cell body. Intersections were measured at 5-micron increments starting 2.5 microns away from the cell body center.

### Statistical analysis

All analyses were completed using GraphPad Prism 9 (GraphPad Software, La Jolla, CA). Data were analyzed using either an unpaired *t*-test, to determine differences between stressed rats treated with either VEH or CNO, or two-way ANOVA with Tukey post-hoc analysis, to compare data in experiments with a 2×2 design assessing CON vs. WS and VEH vs. CNO. Figures displaying Sholl curves were assessed with a three-way ANOVA to determine the effects of stress, drug treatment, and distance. Data were assessed via outlier analysis and any outliers greater than 2x the standard deviation were excluded. Graphs are presented as mea*n* ± standard error (SEM) with an α = 0.05. Significant main effects are reported in the text with significant post hocs reported on the figures.

## Results

### AAV-CD68 is selectively expressed in microglia

**Fig. 2:**
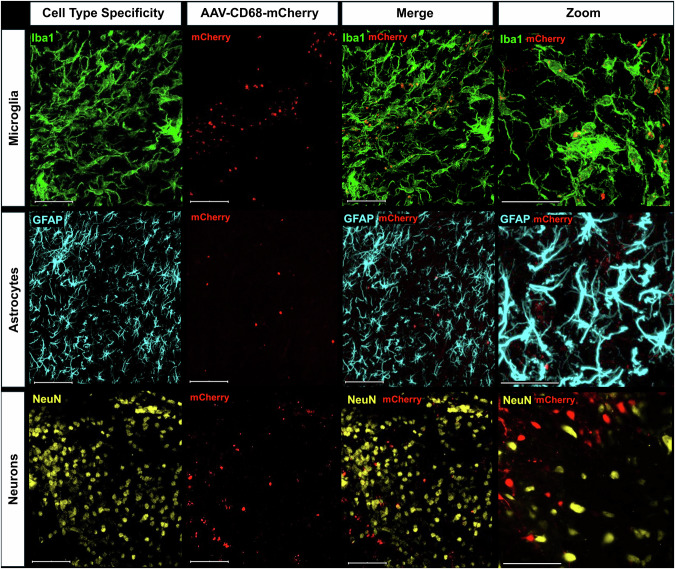
Specificity of viral targeting of microglial cells. Brain slices collected from rats with LC targeted microglial pAAV1 CD68-hM4D(Gi)-mCherry virus injections were stained for Iba1 (microglia, green, row 1), GFAP (astrocytes, blue, row 2), and NeuN (neurons, yellow, row 3). mCherry was visualized to selectively colocalize with Iba1 positive cells and colocalization was lacking with both astrocytes and neurons. Furthermore, mCherry was not observed outside of the LC region.

Experiment #1 used brain tissue collected from female rats ~28 days following intra-LC viral infusion. The CD68 promoter allows selective insertion of G_i_ receptors onto microglial cells, and this virus has been previously characterized for microglial localization and inhibition in the spinal cord [30, 36]. Confocal analysis of these immunofluorescence-stained tissue sections revealed microglial cell type specificity of viral expression with selective colocalization of mCherry with Iba1+ microglia (Fig. 2**Row 1**) and mCherry overlap absent in astrocytes (Fig. 2**Row 2**) and neurons (Fig. 2**Row 3**).

### Chemogenetic inhibition impacts stress-evoked behaviors and cytokine levels

**Fig. 3:**
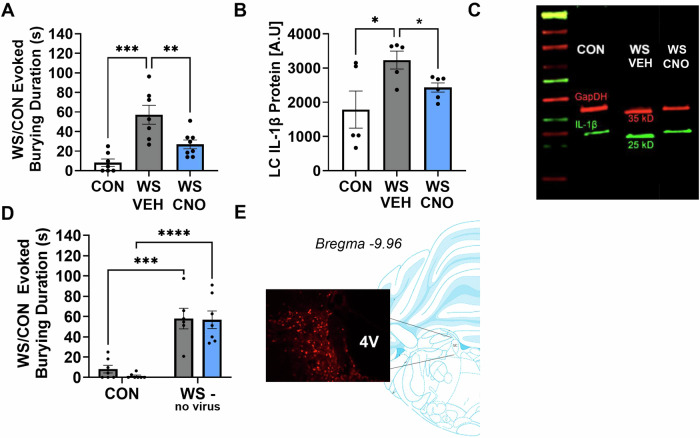
Chemogenetic mediated suppression of microglial reactivity prevented the behavioral and neuroimmune responses to acute WS. **A** CNO treatment in rats administered the inhibitory microglial DREADDs virus in the LC reduced the burying response typically observed in response to repeated witness stress (WS). **B** CNO engagement of the inhibitory DREADD during stress prevented the WS-induced accumulation of the proinflammatory cytokine IL-1β in the LC. **C** Representative Western Blot lanes displaying IL-1β labeling (green) and the housekeeping protein to which it was normalized (GapDH, red). **D** In surgically naïve rats with no viral administration, CNO treatment alone did not lead to any alterations in burying behavior in WS exposed females. **E** Brain atlas location reflecting viral localization and expression which was restricted to the LC. *p < 0.05, **p < 0.008, ***p < 0.0002, ****p < 0.0001.

In accordance with our previous studies [24, 25, 37], WS exposure was associated with significantly heightened burying behavior during acute stress (Fig. 3A, one way ANOVA *F*_*2,19*_ = 14.44, *p* = 0.0002; WS + VEH vs. CON + VEH, *p* = 0.0001). However, if females were treated with CNO one hour prior to WS, this effect was diminished and this group displayed significantly less burying in response to the stressor at levels comparable with controls (Fig. 3A, WS + VEH vs. WS + CNO, *p* = 0.008). One rat in the WS + VEH group was excluded from analysis due to sickness behavior present during the video recording. Similarly, exposure to acute WS evoked an increase in levels of IL-1β within the LC (*p* = 0.0214) an effect that was diminished by DREADD activation with CNO (Fig. 3B, one way ANOVA *F*_*2,13*_ = 4.396, *p* = 0.0348, WS+VEH vs. WS + CNO *p* = 0.0414, WS+CNO vs. CON *p* = 0.3808). Representative Western Blot lanes depicting all treatment groups are presented in Fig. 3C and full blots are presented in the Supplement (Figure S3). A separate group of rats were assessed for the behavioral effects of CNO in the absence of DREADD virus administration. There was a significant effect of stress (Fig. 3D, F1,23 = 62.17, *p* < 0.0001) since WS exposed females treated with VEH displayed significantly higher levels of burying when compared to controls (*p* = 0.0002). Importantly, there was no effect of CNO on burying when the DREADD virus was absent, and WS+CNO-no virus treated rats displayed significantly higher levels of burying when compared to controls (*p* = 0.0001), similar to the WS+VEH-virus group (*p* > 0.99). One animal from the WS+VEH-no virus group was excluded from behavioral analysis due to a missing video file. Brains were collected from all rats and virus localization to the LC was confirmed, with a representative image displaying the optimal atlas localization and LC virus spread in Fig. 3E.

### Chemogenetic suppression of microglial reactivity prevents LPS-evoked microglial retraction

**Fig. 4:**
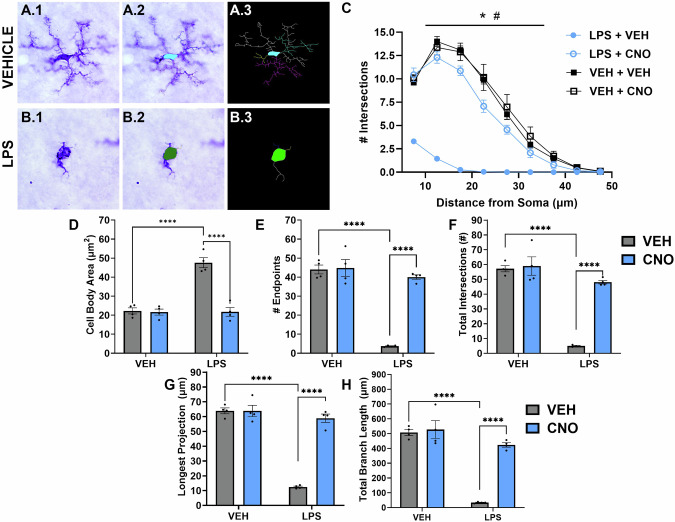
Chemogenetic mediated suppression of microglial reactivity prevented systemic LPS-induced effects on microglial morphology. Brain slices stained with the Iba1 marker of microglia (**A.1**, **B.1**) were processed with Neurolucida software such that the cell bodies and projections were traced (**A.2**, **B.2**) and a skeletonized image of each cell was processed with a Sholl analysis (**A.3**, **B.3**). **C** Sholl curves were calculated using the number of projections that intersected increasing distances from the cell body and showed that, while vehicle treated rats exhibited highly complex microglia with high numbers of intersections around the cell body, LPS treatment reduced the morphological complexity of these cells as expected. Importantly, CNO treatment prior to LPS suppressed microglial reactivity, resulting in higher complexity of these cells, a morphological characteristic indicative of less reactive cells. Additional markers of microglial morphology state indicated that LPS increased cell body area (**D**) and decreased the number of endpoints (**E**) total number of intersections (**F**) length of the longest projection (**G**) and total branch length (**H**) all effects that were reversed with CNO treatment. ^#^*p* < 0.0001 VEH vs. WS, *p < 0.0001 WS vs. CNO, ****p < 0.0001.

Sholl analysis of microglia from female rats treated with either VEH or LPS followed by VEH or CNO (one hour following LPS) revealed a significant difference between the groups (three-way ANOVA, distance x LPS x CNO interaction, *F*_*8108*_ = 17.63, *p* < 0.0001) such that systemic LPS administration resulted in a significantly lower number of intersections at all distances from the cell body when compared to VEH treated rats (Fig. 4C, significant distance x LPS interaction, *F*_*8108*_ = 27.49, *p* < 0.0001; significant LPS effect, *F*_*1,08*_ = 387.60, *p* < 0.0001) that was prevented with CNO administration (significant distance x CNO interaction, *F*_*8108*_ = 14.44, *p* < 0.0001; significant CNO effect, *F*_*1108*_ = 193.50, *p* < 0.0001). Additional measurements revealed an overall reactive cellular morphology in LPS treated rats that was not present with CNO treatment, with a significantly larger cell body area (Fig. 4D, LPS effect *F*_*1,12*_ = 37.70, *p* < 0.0001), lower number of projection endpoints (Fig. 4E, LPS effect *F*_*1,12*_ = 77.48, *p* < 0.0001), decreased total intersections (Fig. 4F, LPS effect *F*_*1,12*_ = 89.70, *p* < 0.0001), shorter projections (Fig. 4G, LPS effect *F*_*1,12*_ = 120.20, *p* < 0.0001), and a lower total projection length (Fig. 4H, LPS effect *F*_*1,12*_ = 79.57, *p* < 0.0001) when compared to controls, and all microglial ramification measures were all reversed with CNO treatment (CNO effect, cell body area *F*_*1,12*_ = 40.92, *p* < 0.0001, number of endpoints *F*_*1,12*_ = 52.46, *p* < 0.0001, total intersections *F*_*1,12*_ = 45.53, *p* < 0.0001, projection length *F*_*1,12*_ = 81.48, *p* < 0.0001, and total projection length *F*_*1,12*_ = 39.92, *p* < 0.0001).

### DREADD activation prevents WS-induced changes in microglial ramification

**Fig. 5:**
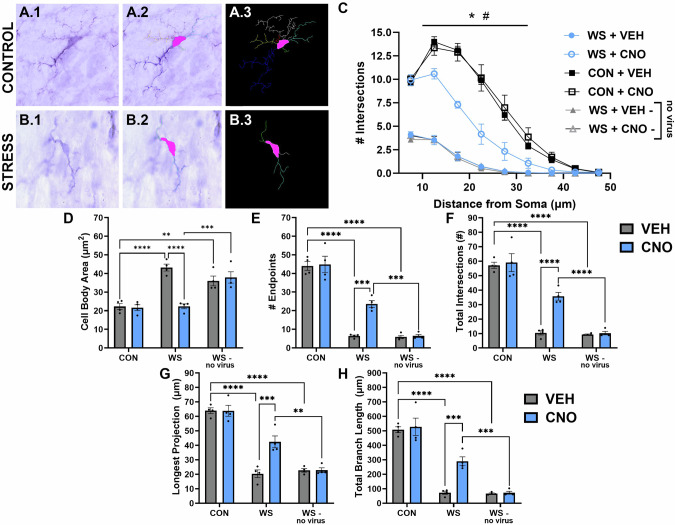
Exposure to witness stress impacted morphological markers of microglial reactivity which were reversed with CNO-mediated DREADD activation. Automated computer processing of microglial ramification characteristics was established using Neurolucida software to trace the cell body area and projections of microglial cells within the LC (**A.1**, **B.1**). A skeletonized image of each cell body and projections (**A2**, **B2**) were analyzed using a Sholl analysis (**A.3**, **B.3**). Plotting the number of intersections over the distance they occurred from the cell body resulted in the creation of a Sholl curve that showed, while control exposed rats maintained a high level of microglial complexity, WS reduced the morphological complexity of these cells, an effect that was reversed with CNO treatment (**C**). Importantly, the WS effect was maintained in CNO treated rats that did not receive viral infusion, indicating DREADD selectivity and lack of off-target effects of CNO on microglial complexity. These results were consistent across additional measures of microglial morphology state including WS evoked effects on increased cell body area (**D**), reduced number of endpoints (**E**) fewer intersections (**F**) shorter projections (**G**) and less overall branch length (**H**). These WS effects were reversed when CNO was given, but only in rats that had active virus injected into the LC, while CNO did not cause off target effects on microglia morphology when administered to those lacking virus. ^#^*p* < 0.0001 VEH vs. WS, *p < 0.0001 WS vs. CNO, **p < 0.005, ***p < 0.0005, ****p < 0.0001.

Microglial ramification characteristics were assessed with a Sholl analysis to compare across stress and CNO treated groups with and without active virus present (three-way ANOVA, distance x stress x CNO interaction, *F*_*8108*_ = 6.586, *p* < 0.0001). Stressed females that received VEH displayed a fewer number of intersections across the distance from the cell body compared to controls (Fig. 5C, distance x stress interaction, *F*_*8108*_ = 29.67, *p* < 0.0001; stress effect, *F*_*1108*_ = 417.70, *p* < 0.0001). Further, rats with active virus treated with CNO prior to stress exhibited an increase in cellular ramification, with a higher number of intersections across the distance from the cell body more similar to non-stressed controls (distance x CNO interaction, *F*_*8108*_ = 4.778, *p* < 0.0001; CNO effect, *F*_*1108*_ = 62.25, *p* < 0.0001). Importantly, rats that were surgically naïve and without viral infusion did not exhibit differences in microglial morphology characteristics when treated with CNO and were significantly different than those that received active virus (distance x virus interaction, *F*_*8108*_ = 16.25, *p* < 0.0001; virus effect, *F*_*1108*_ = 162.40, *p* < 0.0001). When assessing additional microglial characteristics, WS resulted in a significant increase in cell body area (Fig. 5D, *F*_*2,18*_ = 27.22, *p* < 0.0001), decrease in the total number of branch endpoints (Fig. 5E, *F*_*2,18*_ = 161.50, *p* < 0.0001), decrease in the total number of intersections (Fig. 5F, *F*_*2,18*_ = 138.70, *p* < 0.0001), shorter projections (Fig. 5G, *F*_*2,18*_ = 122.20, *p* < 0.0001), and a lower total length of all projections (Fig. 5H, *F*_*2,18*_ = 126.90, *p* < 0.0001). Importantly, the increase in cell body size and decrease in projection complexity, number, and length were all prevented with CNO administration in rats with the active virus prior to stress exposure (CNO effect, cell body area *F*_*1,18*_ = 14.50, *p* = 0.0013, number of endpoints *F*_*1,18*_ = 11.53, *p* = 0.0032, total intersections *F*_*1,18*_ = 14.55, *p* = 0.0013, longest projection *F*_*1,18*_ = 10.54, *p* = 0.0045, and total projection length *F*_*1,18*_ = 11.47, *p* = 0.0033) which did not occur in those without the active virus (WS + CNO vs. WS + CNO-no virus, cell body area *p* = 0.0007, number of endpoints *p* = 0.0002, total intersections *p* < 0.0001, longest projection *p* = 0.0012, and total projection length *p* = 0.0004).

### Microglial suppression prevents the behavioral effects of repeated WS

**Fig. 6:**
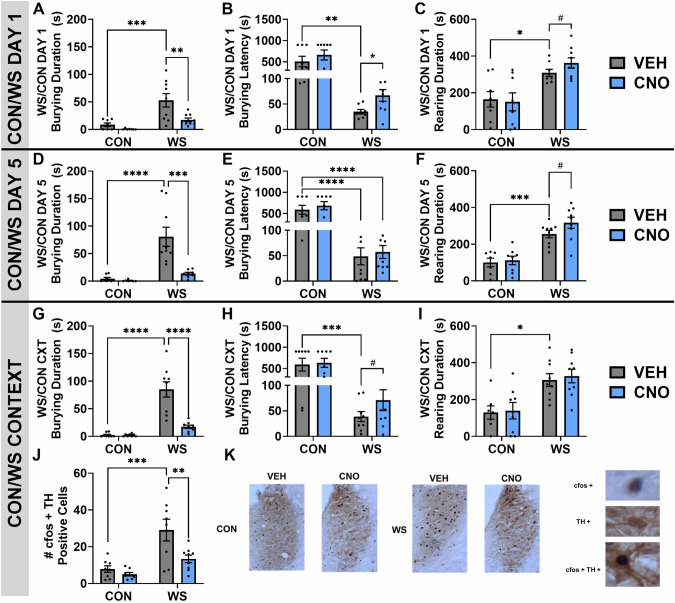
The long-term effects of microglial suppression during stress on behavior and neuronal activity. **A** Exposure to acute WS increased burying when compared to controls and is prevented with suppression of microglial activity in the locus coeruleus (LC) using CNO. **B** CNO treated stressed rats also exhibited a longer latency to begin burying and displayed an increased amount of rearing behavior (**C**). **D** These trends continued with repeated stress where, on the fifth day of WS, stressed rats exhibited heightened burying that was prevented with CNO to suppress microglial reactivity in the LC. Further, while WS exposed females generally exhibited a shorter latency to begin burying when compared to controls, there were no significant differences in the latency to begin burying when comparing CNO treated groups (**E**) while a trend remained with regards to increased rearing behavior in stressed females that received CNO (**F**). To test for hypervigilant behavioral responses following repeated stress, WS exposed females were presented with the stress context to determine stress cue-evoked behavioral responding. WS groups that received vehicle maintained higher levels of burying (**G**) a shorter latency to begin burying (**H**) and an increased amount of rearing (**I**) when compared to controls in response to the stress context. Furthermore, a history of CNO-induced suppression of microglial reactivity during daily WS exposures subsequently had long term implications for behavioral responses when rats were returned to the context (WS/CON CXT exposure). During context re-exposure, rats previously treated with CNO during WS exhibited reduced burying duration and increased latency to start burying. Notably, exposure to the WS context heightened neuronal activity in the LC as evidenced by higher levels of cfos in TH positive cells, an effect that was mitigated by previous microglial suppression using CNO (**J**). Representative examples of cfos and TH staining in each treatment group is presented along with visual criteria for the inclusion as a cfos positive, TH positive, and colabeled cell (**K**). ^#^*p* < 0.075, *p < 0.05, **p < 0.005, ***p < 0.0005, ****p < 0.0001.

Long-term effects of DREADD activation were assessed in rats exposed to repeated stress. There was a significant effect of stress on burying behavior in response to a single stressor (Fig. 6A, main effect of stress, *F*_*1,29*_ = 18.30, *p* = 0.0002), replicating not only the findings shown in Fig. 2A, B, but also our previous publications [24, 25, 37] and additional studies using the WS model [40]. Notably, CNO treatment one hour prior to the stressor diminished the WS-evoked burying response (Fig. 6A, main effect of drug, *F*_*1,29*_ = 9.349, *p* = 0.0048) which replicates the data presented in Fig. 2A. Furthermore, when compared to non-stressed controls, WS exposed females exhibit a shorter latency to begin burying (Fig. 6B, main effect of stress, *F*_*1,29*_ = 41.43, *p* < 0.0001) and an increased duration of rearing (Fig. 6C, *t*_*15*_ = 1.548, *p* = 0.0712), both of which were attenuated by CNO treatment prior to stress (WS+VEH vs. WS+CNO, burying latency *p* = 0.0256, rearing duration *p* = 0.07). No significant levels of freezing were observed in any treatment group (freezing duration, CON+VEH mea*n* = 0 s, CON+CNO mea*n* = 0 s, WS+VEH mea*n* = 1.089 s, WS + CNO mea*n* = 0.038 s, data not shown).

Similar behavioral effects were observed in response to repeated stress. On WS Day #5, burying behavior was heightened in stressed females and attenuated by CNO (Fig. 6D, interaction, *F*_*1,28*_ = 10.06, *p* = 0.0037; main effect of stress, *F*_*1,28*_ = 19.74, *p* = 0.0001; main effect of drug, *F*_*1,28*_ = 12.56, *p* = 0.0014; WS +VEH vs. WS + CNO *p* = 0.0002). While there was no longer an increase in the latency to begin burying in the WS+CNO group (Fig. 6E, main effect of stress *F*_*1,30*_ = 74.46, *p* < 0.0001; WS+VEH vs. WS+CNO *p* = 0.3443), there was still a strong trend towards more exploratory rearing observed in stressed females that were treated with CNO (Fig. 6F, main effect of stress *F*_*1,29*_ = 51.94, *p* < 0.0001; WS+VEH vs. WS+CNO *p* = 0.0542). Additionally, similar to Day 1, there were no notable levels of freezing observed across all groups in response to stress on Day 5 (freezing duration, CON+VEH mea*n* = 0 s, CON+CNO mea*n* = 0 s, WS+VEH mea*n* = 0.863s, WS+CNO mea*n* = 0.80 s, data not shown).

Subsequently, when assessing stress cue-induced hypervigilance during the WS CXT, there was a significant interaction (stress x drug interaction, *F*_*1,30*_ = 19.69, *p* = 0.0001) between stress (main effect of stress, *F*_*1,30*_ = 41.58, *p* < 0.0001) and prior administration of CNO during WS (main effect of drug, *F*_*1,30*_ = 20.05, *p* = 0.0001) in burying duration in response to the stress cues and environment (Fig. 6G), highlighting microglia as controlling the behavioral sensitization to cue-induced burying behaviors. Stressed females previously administered CNO during WS displayed an trend for increased latency to begin burying (Fig. 6H, main effect of stress *F*_*1,30*_ = 40.75, *p* < 0.0001; WS+VEH vs. WS + CNO *p* = 0.087) and no significant differences in the duration of rearing behavior (Fig. 6I, main effect of stress *F*_*1,29*_ = 22.37, *p* < 0.0001; WS + VEH vs. WS+CNO *p* = 0.337) when compared to vehicle treated rats in the WS CXT.

### WS CXT exposure increases LC activity which is prevented by a history of microglial suppression

Brain tissue collected two hours following the start of WS/CON CXT was analyzed for neuronal activity within the LC. There was a significant effect of stress history (*F*_*1,29*_ = 20.92, *p* < 0.0001) and prior CNO treatment (*F*_*1,29*_ = 8.126, *p* = 0.008) on the number of cfos positive TH labeled LC cells in response to the WS CXT (Fig. 6J). Females with a history of WS exhibited a significantly higher level of neuronal activation in the LC in response to the stress cues when compared to controls (*p* = 0.0005). Notably, if rats had been previously exposed to WS and received CNO for suppression of microglial reactivity, this neuronal sensitization to the stress cues was absent (WS + VEH vs. WS + CNO, *p* = 0.0084). Representative images of TH staining in the LC (brown) along with cfos positive cells (black) are shown for all treatment groups in Fig. 6K.

## Discussion

These studies are among the first to utilize site specific microglial-targeted chemogenetics to manipulate neuroimmune activity within the brain and expand our recent evidence that microglia regulate LC-specific hypervigilant behavior resulting from WS in females [25]. These experiments characterized the use of an inhibitory DREADD-expressing virus driven by the CD68 promoter within the LC of female rats and determined that this virus selectively colocalized with microglia, confirming published studies using these techniques in the spinal cord [30, 31]. These findings demonstrate that chemogenetic suppression of microglial reactivity prevented the effects of WS on morphological markers of microglial activation state, reduced WS-evoked inflammatory cytokine production, and prevented WS-induced hypervigilant behaviors both in response to stress and stress cues. Notably, the DREADD-mediated reduction in stress-induced neuroimmune activity during WS was associated with a lower level of CXT-induced neuronal activity in the LC. Taken together, these experiments have characterized the use of a CD68 promoter-driven hM4Di virus for targeting and manipulating microglial reactivity within the rat hindbrain in response to LPS and WS challenges and support the role of LC neuroimmune signaling in the acute and long-term impact of stress in females.

Previous studies by our lab [25, 41, 42] and others [43–45] have highlighted the essential role of neuroimmune signaling in mediating CNS responses to stress [46]. Importantly, the presence of proinflammatory cytokines in the LC leads to activation of the norepinephrine-producing LC cells which can have widespread consequences for downstream stress-sensitive regions [47]. The concentration of cytokines such as IL-1β required to activate neuronal cells is ~1000 fold lower than that required by other cell types [48]. Thus, even modest accumulations of IL-1β in the LC has the capacity to induce robust changes in neuronal activity, as indicated by the current findings. Further, inflammatory-mediated regulation of LC activity has been associated with hypervigilant and depressive-like behaviors [13, 49]. These experiments determined that microglial inhibition impacted behavioral responses to acute and repeated psychosocial stress, and this decrease in hypervigilant behavioral responding also occurred in response to subsequent exposure to the stress cues and environment. The inflammatory consequences of repeated stress have been shown to be sensitized with repeated stress exposure, a phenomenon often referred to as a “dual hit” hypothesis [21, 46]. The neuroimmune signaling and microglial reactivity that occurs in the brain as a result of social stress exposure leads to sensitization, whereby subthreshold stressor exposure in the future, i.e. exposure to the stress context, would therefore lead to an exaggerated inflammatory response [46]. Therefore, by preventing the inflammatory cascade that typically occurs in response to social stress [2, 24, 25, 41, 46] with chemogenetic inhibition of microglial activation, these results suggest that microglial priming and stress-induced sensitization of neuroimmune responding to subsequent stress and stress cues were blocked. Ultimately, we hypothesized that neuroimmune activity within the LC regulates hypervigilant behaviors through its effects on neuronal signaling, which was confirmed with cfos staining. Future experiments utilizing in vivo electrophysiology to directly measure neuronal activity in response to microglial blockade will allow us to further probe the relationship between microglial and neuronal activity in the LC in the female stress response.

These studies identified that LC microglia control the behavioral response to WS and, when microglial reactivity is blunted, WS-evoked burying and rearing behavior are impacted in response to both acute and repeated stress. Notably, neuroimmune signaling in the LC during WS exposure was also critical for the development of the sensitized behavioral burying response that occurs when rats are further exposed to the stress cues and environment. Stressed females display a higher amount of burying along with less rearing behavior, which can be interpreted as an increase in compulsive anxiety-like behavior paralleled by a decrease in exploration. This data is supported by additional studies that have identified distinct LC projections to the central amygdala and medial prefrontal cortex that are responsible for increasing burying and decreasing exploration, respectively, in response to an external threat [14]. When assessing further behaviors that could be presented in response to stressful stimuli, freezing behavior was not observed in response to WS, emphasizing buying responses as the main anxiety-like behavior that is presented in response to this social stressor. While burying itself is not a translational measure, the ethological relevance of burying behavior in the rodent as a perseverative and repetitive behavior represents an important correlate to hypervigilant behaviors observed in humans with stress-related conditions such as PTSD [50, 51]. Further, burying behavior is associated with clinical signs of stress including increased stress hormone signaling and heart rate [25] and is highly dependent on the noradrenergic system [52], all which provide support for construct validity. Additionally, previous experiments from our lab have used translational measures of hypervigilance such as the acoustic startle task which can be completed similarly in rodents and humans. These studies show that rats exposed to WS subsequently develop a higher acoustic startle response [37] and future experiments will be required to determine the impact of LC microglial reactivity on this translational measure of hypervigilance. While the LC is known to regulate hypervigilance and arousal in response to stressful experiences [13], this region also plays a role in conditional fear responding. Interestingly, LC projections are involved in hippocampal-dependent memory underlying the conditioned responses presented during associative fear learning [53]. Furthermore, it has been long established that norepinephrine signaling within the amygdala supports contextual fear learning [54], a response that is escalated in females [55]. Since these experiments involved multiple presentations of the stressor along with exposure to the stress cues, this model of psychosocial stress exposure may involve similar neural mechanisms to those required for contextual associative learning. Further, suppressing microglial reactivity in these studies may reduce LC activity and impair associative learning between stress exposure and stress cues. These questions serve as important future directions that will be investigated to establish the impact of direct microglial modulation in the LC on classical fear conditioning paradigms.

While chemogenetic techniques have been extensively utilized for their ability to impact neuronal signaling [56, 57], this method has only recently begun to be optimized for use in non-neuronal cell types [58–60]. Evidence for the ability of AAVs to transduce microglia has been equivocal, likely due to differences in species, strain, brain region, and viral serotype. However, AAV1 serotypes have been determined to effectively transduce microglial cells across the brain [61]. Further, microglial reactivity, along with the resulting pro-inflammatory cytokine release, has been shown to be regulated through GPCR-mediated mechanisms [62, 63] which supports the use of G_i_ modulation to affect the inflammatory state of these cells. Importantly, this virus has a functional impact on the evoked expression of pro-inflammatory mediators IL-6, IL-1β, and TNFα [30, 31]. Additionally, viral transduction into microglia has been observed to have regional heterogeneity [59, 61], and these are the first experiments to observe a high level of microglial transduction within the LC. Further, species specific differences in microglial genetics, primarily the ~6x higher levels of CD68 RNA observed in resting microglia in the rat versus the mouse [64], may influence differential rates of microglial transduction between the species. One further possible confound when using chemogenetic techniques is the risk for unintended effects of CNO outside of its function at the designed receptors. While higher doses of CNO, such as 10 mg/kg, produce off target effects and yield a high rate of conversion to clozapine, CNO at doses of 1 mg/kg, like those used in the present study, have been shown to have limited, if any, off target effects while plasma levels of clozapine are undetectable in rats [65, 66]. Importantly, these studies confirmed the lack of effect of CNO on behavior or microglial morphology to alleviate these concerns.

In conclusion, these experiments are among the first to site-specifically target microglia within the rat hindbrain for chemogenetic manipulation of neuroimmune signaling in response to stress. These studies confirmed our previous findings establishing LC microglia as a key player in the female stress response [25] and expanded the role of neuroimmune signaling to subsequent behavioral and neuronal responses to stress cues. Taken together, these experiments characterized the use of chemogenetics to alter microglial reactivity in the LC and support the role of LC neuroimmune signaling as a key mechanism in the female stress response. Subsequent experiments will examine the functional relationship between stress-evoked microglial responses, neuronal signaling, and resulting NE output from the LC, as well as assessing sex differences. Overall, the current results highlight the potential for targeting neuroimmune signaling in the treatment of stress-related comorbidities in females.

## Supplementary information

Supplemental Materials

## Data Availability

The datasets generated and analyzed for the current study are available from the corresponding author on reasonable request.
