# Supplementary material for: The functional role of locus coeruleus microglia in the female stress response
Source: Mol Psychiatry. 2025 Apr 5;30(9):3925–36. doi: 10.1038/s41380-025-02971-9 (PMC12339389; doi:10.1038/s41380-025-02971-9)
Supplement: Supplementary file 1 — Supplemental Materials [file 41380_2025_2971_MOESM1_ESM.docx]

**Supplementary Materials for:** **The role of locus coeruleus neuroimmune signaling in the response to social stress in female rats**

**Supplementary Figure 1.** Virus placement localization within the LC for all treatment groups.


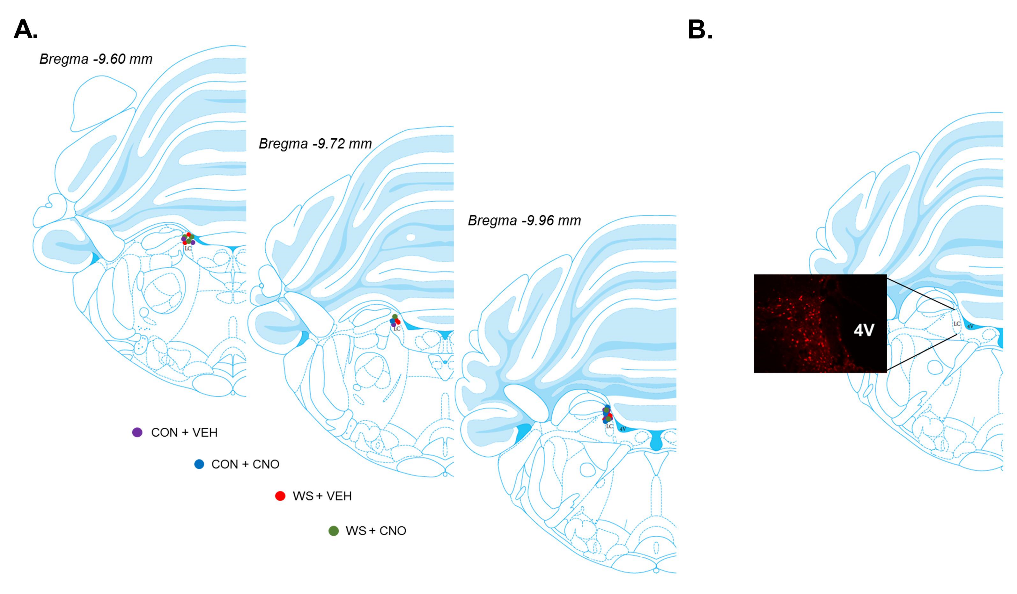


**Supplementary Figure 2.** The location of micropunches taken to include the locus coeruleus.


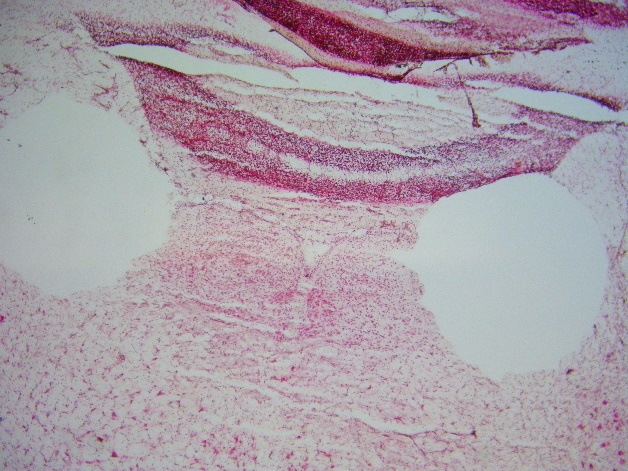


**Supplementary Figure 3.** Full Western Blot images for Figure 3B with GapDH (red) and IL-1β (green).

**
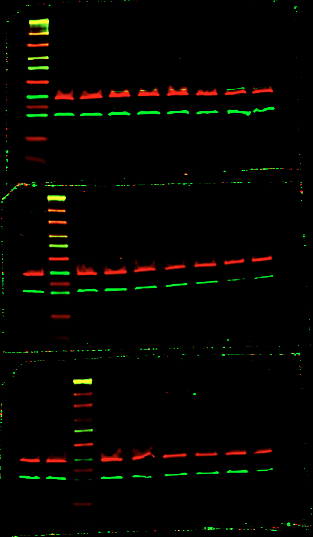
**
